# How Diffusivity, Thermocline and Incident Light Intensity Modulate the Dynamics of Deep Chlorophyll Maximum in Tyrrhenian Sea

**DOI:** 10.1371/journal.pone.0115468

**Published:** 2015-01-28

**Authors:** Davide Valenti, Giovanni Denaro, Bernardo Spagnolo, Fabio Conversano, Christophe Brunet

**Affiliations:** 1 Dipartimento di Fisica e Chimica, Università di Palermo, Group of Interdisciplinary Theoretical Physics and Consorzio Nazionale Interuniversitario per le Scienze Fisiche della Materia, Unità di Palermo, Palermo, Italy; 2 Istituto Nazionale di Fisica Nucleare, Sezione di Catania, Catania, Italy; 3 Radiophysics Department, Lobachevsky State University, Nizhniy Novgorod, Russia; 4 Stazione Zoologica Anton Dohrn, Naples, Italy; CNRS, FRANCE

## Abstract

During the last few years theoretical works have shed new light and proposed new hypotheses on the mechanisms which regulate the spatio-temporal behaviour of phytoplankton communities in marine pelagic ecosystems. Despite this, relevant physical and biological issues, such as effects of the time-dependent mixing in the upper layer, competition between groups, and dynamics of non-stationary deep chlorophyll maxima, are still open questions. In this work, we analyze the spatio-temporal behaviour of five phytoplankton populations in a real marine ecosystem by using a one-dimensional reaction-diffusion-taxis model. The study is performed, taking into account the seasonal variations of environmental variables, such as light intensity, thickness of upper mixed layer and profiles of vertical turbulent diffusivity, obtained starting from experimental findings. Theoretical distributions of phytoplankton cell concentration was converted in chlorophyll concentration, and compared with the experimental profiles measured in a site of the Tyrrhenian Sea at four different times (seasons) of the year, during four different oceanographic cruises. As a result we find a good agreement between theoretical and experimental distributions of chlorophyll concentration. In particular, theoretical results reveal that the seasonal changes of environmental variables play a key role in the phytoplankton distribution and determine the properties of the deep chlorophyll maximum. This study could be extended to other marine ecosystems to predict future changes in the phytoplankton biomass due to global warming, in view of devising strategies to prevent the decline of the primary production and the consequent decrease of fish species.

## Introduction

The study of the mechanisms responsible for the distribution and dynamics of phytoplankton populations represents one of the most urgent challenges for marine ecological modeling, due to emergent problems such as global warming and reduction of the biomass production in marine ecosystems [1]. Indeed, the variations in the growth of fish species observed in the oceans are mainly explained by changes in the chlorophyll concentration, which is a marker of the presence of phytoplankton populations [1–10].

During last decades specific applications of population dynamics and theoretical models allowed to describe the behaviour at the group level, and to obtain the spatio-temporal distributions of phytoplankton populations in aquatic environments. Moreover, in recent works [11–17], the effects of the spatial heterogeneity of the limiting factors on the diversity of phytoplanktonic populations, along the water column, has been investigated. However, authors have always reproduced the vertical profiles of chlorophyll concentration without taking in account the seasonal changes of environmental variables [16, 17], such as light intensity, depth of thermocline and vertical turbulent diffusivity.

In a marine ecosystem, the shape of vertical phytoplankton profiles depends on the spatial behaviour of two limiting factors [11, 18–20], i.e. light intensity and nutrient concentration, which allow the photosynthesis process within phytoplankton cells. Specifically, the reduction of the light intensity, as a function of depth, associated with an opposing gradient of nutrients allows to maintain a positive net growth rate only within the production layer. It is worth noting that, despite other quantities such as turbulence and mixing play a key role on the spatio-temporal behaviour of the phytoplankton populations, they can not be considered limiting factors because they do not contribute directly to the photosynthesis process. In general, the limiting factors contribute to select different groups along the water column [11, 12, 17, 21–23], even if both the position of the favorable layer and the magnitude of concentration peak, for each phytoplankton population, depend on biological and physical parameters [18, 24, 25]. Specifically, light and nutrient half-saturation constants, maximum growth rate and mortality determine the boundaries of the production layer, while its width and the peak magnitude are connected to the vertical turbulent diffusivity and the swimming velocity of each population. Because the vertical distributions of limiting factors change continuously during the year, due to seasonal variations of environmental variables, the production layer for every phytoplankton populations is highly variable [22].

In order to analyze the seasonal dynamics of phytoplankton, we recall that marine ecosystems are an example of complex systems, which are open systems characterized by nonlinear interactions among their parts and external perturbations due to environmental variables. Complex systems are present in different scientific areas ranging from condensed matter to econophysics and biophysics [26–50]. In the ecosystem investigated here, the continuous variability of physical parameters, observed in real data, indicates that in order to reproduce the seasonal dynamics of phytoplankton populations it is necessary to perform a study which takes into account relevant physical and biological issues, such as the effects of variable mixing in the upper layer, the competition between groups occupying different layers of the water column, and the dynamics of non-stationary deep chlorophyll maxima.

In this work, we present a theoretical approach to model the spatio-temporal behaviour of phytoplankton abundance distribution at group level. Here, phosphorus is the nutrient component playing the role of limiting factor for the growth of the phytoplankton populations [17, 51, 52]. The site investigated represents an ideal habitat to analyze how ecosystem hydrodynamics affects the phytoplankton distribution [53]. Indeed, in this area, meso-scale hydrodynamic structures modify seasonally the vertical profiles of nutrients and light environment [54], and thus indirectly control both the biomass primary production and phytoplankton composition [53, 55–57].

The theoretical approach is based on a reaction-diffusion-taxis model, which allows to analyze and reproduce the vertical profiles of *chlorophyll a* concentration obtained from data sampled in a site of the Tyrrhenian Sea (along the south-western Italian coast of the Mediterranean Sea), characterized by oligotrophic conditions, during different oceanographic surveys in the period from 24 November 2006 to 9 June 2007. In particular, the competition between phytoplankton populations for light and nutrient (phosphorus) has been modeled by using a system of coupled reaction-diffusion-taxis equations. The model is able to reproduce the spatio-temporal behaviour of five populations belonging to the picophytoplankton fraction, i.e. planktonic groups whose linear size is less than 3 *μ*m. This fraction is formed by groups belonging to two different domains, i.e. picoprokayotes and picoeukaryotes [58–60], and takes in account, on average, about 80% of the total *chlorophyll a* (*chl a*) and *divinyl chlorophyll a* (*Dvchl a*) in Tyrrhenian Sea.

In order to guarantee the coexistence of the five populations along the water column, suitable values of biological and environmental parameters have been set in the model. Moreover, the influence of the upper mixed layer (UML) on spatial configuration of picophytoplankton groups is taken into account by assuming larger values of vertical turbulent diffusivity in the UML and much smaller values in the deeper layers.

As a first step, we analyze the role of the upper mixed layer, which determines the shape of the vertical profiles of nutrient concentration and cell concentration for all phytoplankton populations [22, 61]. The UML is generated by variable mechanical perturbations of the water surface, e.g. by wind and storms, and is characterized by the mixing intensity and the layer thickness [62–67], which change periodically as a function of the time. As a consequence, the abundance peak of each phytoplankton group can be localized, alternatively, closer to the water surface or in deeper layers, during the same year. Afterwards we study, in deeper layers, the behaviour of the vertical turbulent diffusivity, whose values change according to the seasonal changes in mean velocities of the marine currents [68]. As a third step, in order to get conditions as much as possible similar to the marine ecosystem investigated, we take into account the effects of the two main limiting factors, i.e. light intensity and phosphorus concentration, simulating the spatio-temporal dynamics of the phytoplankton concentration, and obtaining biomass vertical profiles at different periods of the year. Finally, in order to compare theoretical results with field observations, the picophytoplankton cell concentrations, expressed in *Cells/m^3^*, are converted in *chl a* and *Dvchl a* concentrations, expressed in *μg*/ *dm^3^*, by using the experimental cellular content measured by Morel and the conversion curves obtained by Brunet et al. [69, 70]. Goodness of agreement between theoretical chlorophyll distributions and corresponding experimental profiles is evaluated for all seasons investigated, by performing statistical checks based on *χ^2^* test.

### Environmental data

**Figure 1:**
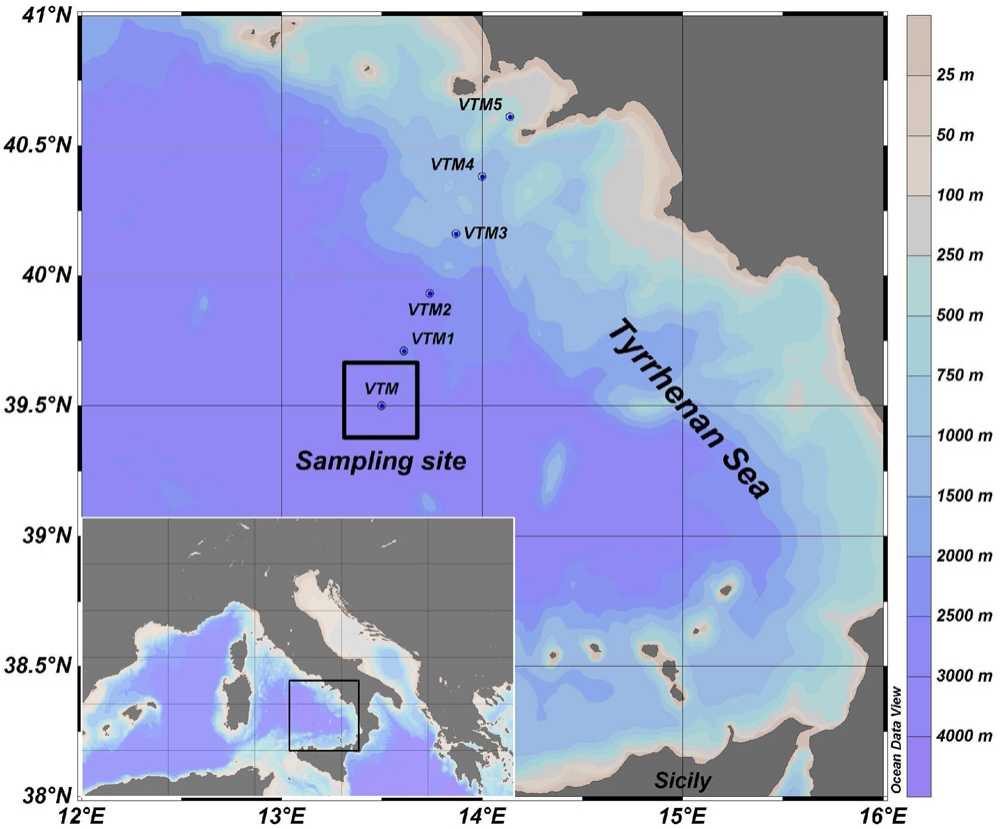
Location of the sampling site (39° 30′.00 N, 13°30′.00 E) where the experimental data were collected. (Courtesy of Ribera d’Alcalà et al., 2009 (Ref. [71])).

The experimental data, analyzed in this work, were collected in the period from 24^th^ November 2006 to 9^th^ June 2007 in a sampling site (39° 30.00′ N,13°30.00′ E) localized in the middle of the Tyrrhenian Sea (Fig. 1). This is a hydrological stable area of Mediterranean Sea, where oligotrophic waters are mainly populated by picophytoplankton groups during the whole year. These groups are located in Modified Atlantic Water (MAW), i.e. the upper layer of the water column of Mediterranean Sea (from the surface down to 200 m). The MAW is placed above the Levantine Intermediate Water (LIW), i.e. the intermediate layer of the Mediterranean basin, and corresponds to the euphotic zone of the water column.

**Figure 2:**
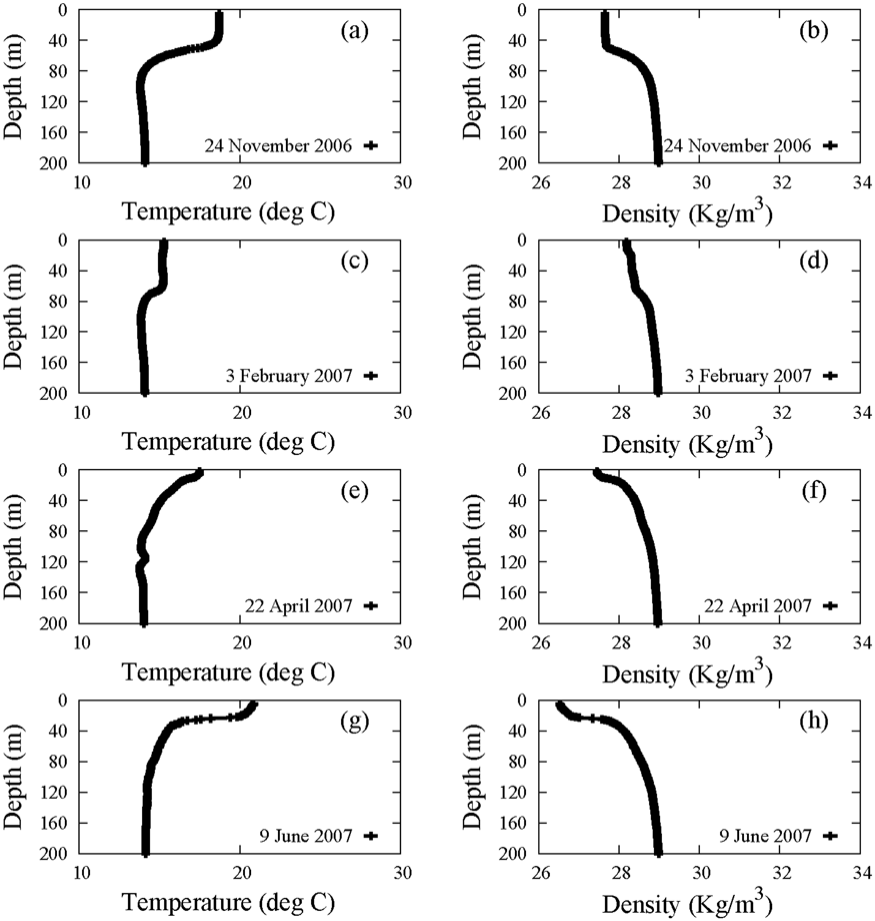
Profiles of temperature (panels a, c, e, g) and density (panels b, d, f, h) acquired in the sampling site (39° 30′.00 N, 13° 30′.00 E). Data were collected during four oceanographic cruises: VECTOR-TM1, 24 November 2006 (panels a, b); VECTOR-TM2, 3 February 2007 (panels c, d); VECTOR-TM3, 22 April 2007 (panels e, f); VECTOR-TM4, 9 June 2007 (panels g, h). The black lines have been obtained by connecting the experimental points corresponding to samples distanced of 1 meter along the water column. The total number of samples measured in the site is *n* = 196 for VECTOR-TM1, *n* = 198 for VECTOR-TM2, *n* = 199 for VECTOR-TM3, and *n* = 196 for VECTOR-TM4.

In order to study the seasonal variability of physical parameters and phytoplankton concentrations, the experimental data were acquired at four different times (seasons) of the year, during four different oceanographic cruises (VECTOR-TM1, November 2006; VECTOR-TM2, February 2007; VECTOR-TM3, April 2007; VECTOR-TM4, June 2007) performed on board of the R/V Urania of the Italian National Research Council (CNR). In addition, a more accurate estimate of vertical turbulent diffusivity for the whole year was obtained by taking into account the vertical profiles of physical parameters (experimental results here not shown) acquired during other three oceanographic cruises (VECTOR-TM6, January 2009; VENUS1, August 2010; TYR01, October 2010). In all these oceanographic surveys, similar sampling strategies and methodologies were used. In particular, vertical profiles of temperature, salinity and density were acquired by using a CTD probe equipped with a fluorescence sensor, which measured total chlorophyll concentrations. The vertical distributions of water temperature and density, measured in the MAW, are shown in Fig. 2. In particular, vertical distributions of water temperature were used to study the displacement of the thermocline along the water column as a function of time, and to determine the thickness of the upper mixed layer (UML). Analogously, vertical distributions of water density were used to calculate the buoyancy frequency of Brunt-Väisälä, necessary to estimate the vertical turbulent diffusivity in UML for all periods of the year investigated.

The experimental profiles shown in Fig. 2 indicated the presence of an upper mixed layer, from the surface down to the thermocline, where the temperature was characterized by values higher than those observed in deeper layers. Conversely the density, characterized by low values within the upper mixed layer, increased below the thermocline. Finally, we note that experimental data indicated an increase of the thermocline depth during fall and early winter up to reach the maximum value in February at a depth of 69 m. Vice versa, the depth of thermocline decreased during early spring up to reach the minimum value in April at a depth of 13 m, maintaining shallower depths during the summer.

Nitrate, nitrite, silicate and phosphate concentrations, collected in polyethylene vials and stored at -20 *ºC*, were determined by using classical methods of measurement [71–73].

### Phytoplanktonic data

Phytoplankton biomass can be estimated using chlorophyll as a proxy (chlorophyll and divinyl chlorophyll). The contribution of each phytoplankton group to the total amount of chlorophyll is based on the experimental estimation of cellular *chlorophyll a* content [69, 70, 74] and the theoretical abundance obtained from the model [16, 17]. Moreover, we performed the high-performance liquid chromatography (HPLC) analysis of the bottle samples collected approximately at the same depths (0, 25, 50,. . . 100 meters) during the oceanographic surveys. The results of HPLC analysis showed that the average amount of phytoplankton fractions obtained in the sampling site of Tyrrhenian Sea during the period investigated (from 24 November 2006 to 9 June 2007) were similar to those obtained from the bottle samples collected in the Strait of Sicily in 1997 [74].

In general, phytoplankton community could be divided into three main size fractions [59, 75]: pico- (*<3μm*), nano- (3–20*μm*) and micro-phytoplankton (> 20 *μm*).

In the Tyrrhenian Sea, the picophytoplankton fraction amounts in average to about 80% of the total *chl a* and *Dvchl a*, and is dominated by two groups: picoprokaryotes and picoeukaryotes. The picoprokaryotes domain is composed of two genera of cyanobacteria, i.e. Synechococcus and Prochlorococcus, while picoeukaryotes domain is mainly represented by haptophytes and pelagophytes [72, 76, 77]. Finally, diatoms, cryptophytes and dinophytes are present in traces.

The nano- and micro-phytoplankton fraction accounts for about 20% of the total *chl a* and *Dvchl a* on average, and is mainly represented by haptophytes, pelagophytes and diatoms. This fraction is poorly present in DCM, and is almost uniformly distributed along the water column.

In this work, we consider the spatio-temporal dynamics of picophytoplankton fraction, focalizing on five groups. In particular, close to the water surface a prevalence of Synechococcus on the others groups is observed, with Prochlorococcus concentration remaining constant with depth. Conversely, in intermediate layers Prochlorococcus prevails respect to other populations [70]. In particular, the ratio between the cell concentration of Prochlorococcus and Synechococcus shows a significant increase as a function of depth. The vertical profiles of Prochlorococcus concentration have a bimodal distribution in the intermediate layers of the water column [70, 77], indicating the coexistence of two ecotypes of this genus: high light-adapted (HL-) ecotype and low light-adapted (LL-) ecotype. However, the spectral light requirements of the two Prochlorococcus ecotypes do not lead to a marked vertical separation [18], even if the former ecotype is mainly localized in the upper part of the euphotic zone between the surface water and 90 m of depth, while the latter is mostly present at depths greater than 50 m [70, 76, 77]. Moreover, we observe that the growth rate and the average cell concentration of LL ecotype are lower than those of HL ecotype in Tyrrhenian Sea. Therefore, in the site investigated the Prochlorococcus HL-ecotype prevails on the LL-ecotype.

It is worth to underline that heterogenous composition is also a feature of the picoeukaryotes domain. In particular, in a previous work [60], it has been shown how a clear segregation along the water column of classes belonging to the picoeukaryotes domain is present in Mediterranean Sea. Specifically, Brunet et al. have found that haptophytes are more abundant in shallower layers of DCM, while pelagophytes dominate deeper layers [70, 74].

On this basis, in this work we model the spatio-temporal dynamics of Synechococcus, Prochlorococcus HL-ecotype, Prochlorococcus LL-ecotype, haptophytes, and pelagophytes, which are the populations mainly present along the water column. We note that, due to different localizations along the water column, from a theoretical point of view it is possible to predict, in the site investigated, the coexistence conditions for all these groups [12, 14, 16, 17, 22].

In view of obtaining a comparison between theoretical results and experimental data, we recall that phytoplankton populations are usually identified and counted by flow cytometry using their scattering and autofluorescence properties [74]. In general, the presence of *chlorophyll a* (*chl a*) or *divinyl chlorophyll a* (*Dvchl a*) molecules in phytoplankton cells allows to distinguish them from non-photosynthetic particles, and to estimate the abundance of phytoplankton populations through the conversion curves. Specifically, the analysis of pigments showed the presence of *chl a* in all phytoplankton groups except Prochlorococcus, which can be distinguished from the rest of the phytoplankton community because of its content of *Dvchl a*, whose molecular structure is almost identical to that of *chl a*.

The analysis of bottle samples indicates that Synechococcus contributes to more than 20% of the total chlorophyll concentration on average in the Mediterranean Sea [70, 74]. In particular, the *chl a* cellular content of Synechococcus does not show appreciable variations in the shallower layers, even if its average values change in different areas of world. Since the *chl a* experimental cellular content of Synechococcus in Tyrrhenian Sea has not been given in previous works, here we used the content measured by Morel in Mauritania coast, whose value was fixed equal to 2 fg *chl a* cell^-1^ [69].

In the Strait of Sicily, Prochlorococcus and picoeukaryotes dominate deeper layers and contribute equally to the picophytoplankton biomass in terms of *chl a* and *Dvchl a* concentrations in DCM, even if Prochlorococcus are numerically more abundant than the picoeukaryotes [70]. In particular, the bottle samples collected in Mediterranean Sea showed that the cellular content of *chl a* and *Dvchl a* increases in picoeukaryotes and Prochlorococcus with decreasing light conditions [70, 78]. More specifically, in the experimental analysis performed with light intensity ranging from a maximum value near the surface to less than 1% of the incident light intensity below the euphotic zone (approximately 100 m of depth), picophytoplankton communities display an increases in cell size and pigment content, which generally occur below the upper mixed layer [79]. In fact, the *Dvchl a* cellular content of the total Prochlorococcus, including both ecotypes, ranges between 0.25 and 2.20 fg *Dvchl a* cell^-1^ along the water column, with a mean value exponentially increasing with depth [70]. On the other side, the mean *chl a* cellular content of the picoeukaryotes ranges between 10 fg *chl a* cell^-1^ in sea surface and 660 fg *chl a* cell^-1^ in deeper layers [70]. This conversion curve has been used to estimate *chl a* concentrations due to the presence of both picoeukaryotes groups investigated, i.e. haptophytes and pelagophytes. The experimental results showed, for depth greater than 100 m, a considerable decrease of the cell concentration of picophytoplankton, due to the dramatic diminution of the light intensity. This strong reduction of cell concentration below the euphotic zone allows to exploit the conversion curves, introduced by Brunet et al. [70], also for deeper layers, describing, without significative errors, the increase in pigment content per cell. In general, these curves are not used for picophytoplankton groups localized close to the surface water. Therefore, because Synechococcus are placed in shallower layers of the water column, the *chl a* cellular content of this genus has been fixed constant in agreement with the experimental results obtained by other authors [69, 78, 80].

**Figure 3:**
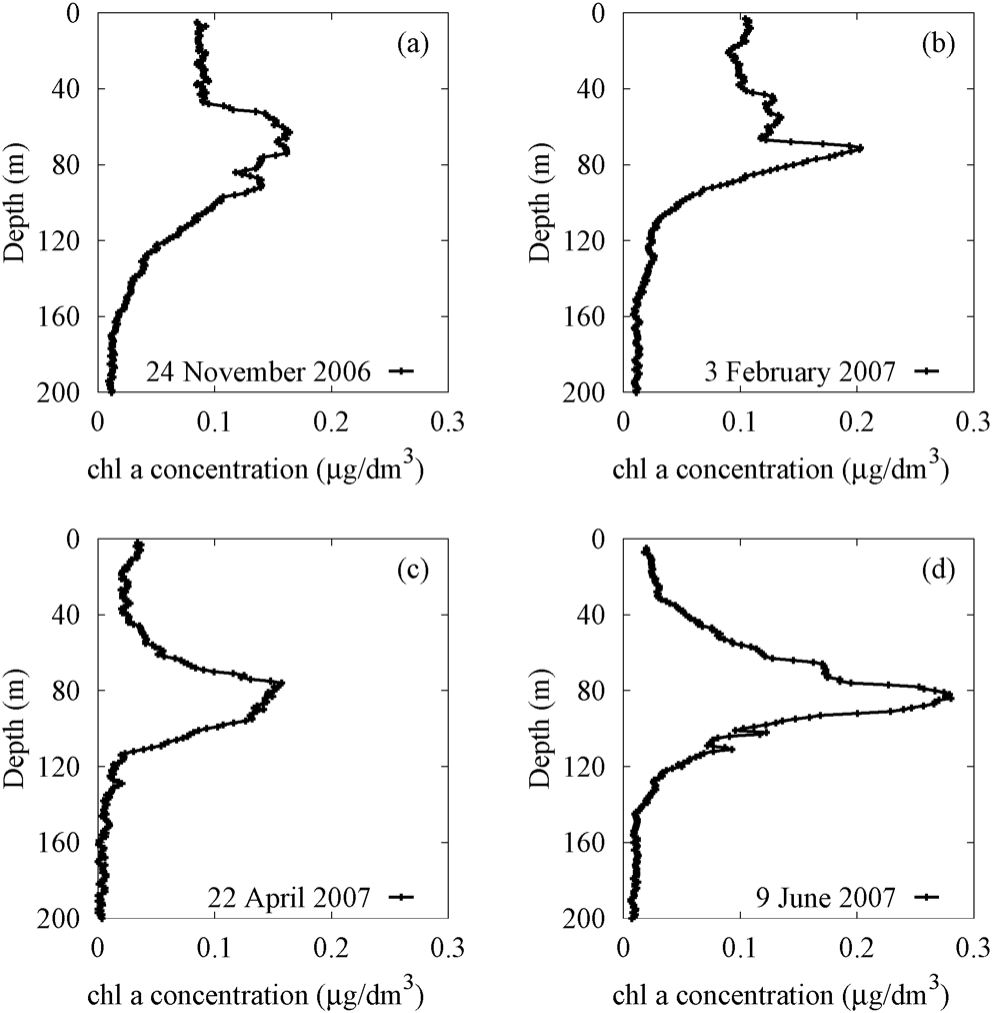
Profiles of *chl a* concentration acquired in the sampling site (39° 30′.00 N, 13° 30′.00 E). Data were collected during four oceanographic cruises: VECTOR-TM1, 24 November 2006 (panel a); VECTOR-TM2, 3 February 2007 (panel b); VECTOR-TM3, 22 April 2007 (panel c); VECTOR-TM4, 9 June 2007 (panel d). The black lines have been obtained by connecting the experimental points corresponding to samples distanced of 1 meter along the water column. The total number of samples measured in the site is *n* = 196 for VECTOR-TM1, *n* = 198 for VECTOR-TM2, *n* = 199 for VECTOR-TM3, and *n* = 196 for VECTOR-TM4.

The vertical profiles of *chl a* concentration, measured at different times of the year in the site analyzed in this work (localized along the south-western Italian coast of the Tyrrhenian Sea with coordinates 39° 30.00′ N,13° 30.00′ E), show a nonmonotonic behaviour (see Fig. 3) characterized by the presence of DCM below the thermocline. The depth of DCM, i.e. the position along the water column in which the total *chl a* concentration reaches the maximum value, ranges between 63 and 84 m, while its magnitude, shape and width change as a function of the time. In particular, the *chl a* concentration in DCM reaches the maximum value (0.28 *μg* l^-1^) in late spring, when a limited mixing generates a strong stratification of the water masses. The same phenomenon causes, in autumn, a decrease of *chl a* concentration in deeper layers. This behaviour can be related with a decrease of nutrient concentration in the DCM due to reduced mixing during summer and early fall. Finally, an increase of the width of the DCM is observed in late fall and winter, when the vertical turbulent diffusivity increases along the whole water column.

The experimental findings show that the *chl a* concentrations take on almost uniform values in the upper mixed layer in all profiles studied (see Fig. 3). However, the biomass concentration in UML changes during the year, showing a maximum of *chl a* concentration (0.10 *μg* l^-1^) in winter. This behaviour is a direct consequence of the variability of the vertical turbulent diffusivity, which reaches a maximum in February in the UML, taking on low values in other periods of the year.

Finally, in order to localize the exact position of the production layer for all picophytoplankton communities, we studied the results of the HPLC analysis of the bottle samples acquired along the water column in the site investigated (Tyrrhenian Sea), during the oceanographic surveys. The *chlorophyll a* and *divinyl chlorophyll a* concentrations measured by the HPLC analysis (experimental results here not shown) were compared with those obtained by the fluorescence sensor, showing a good qualitative agreement.

## Methods

In this section the spatio-temporal behaviour of the five picophytoplankton populations is analyzed by using a mathematical model, consisting of a system of partial derivative equations. The analysis is performed by taking into account the intraspecific competition of each group for light and nutrients within the euphotic zone of the water column, where physical and biological conditions are favourable for the phytoplankton growth.

**Figure 4:**
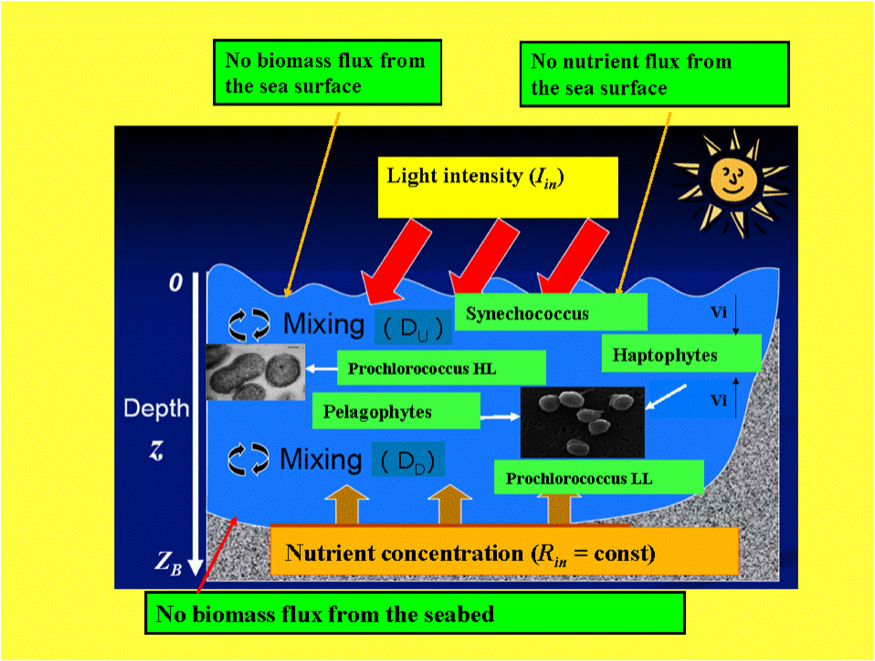
Scheme of the mechanism responsible for the phytoplankton distribution (modified from original figure by Alexey Ryabov). Inset: (a) Prochlorococcus PCC 9511 (courtesy of Rippka et al., 2000 (Ref. [95])), (b) Micromonas NOUM17 (courtesy of Augustin Engman, Rory Welsh, and Alexandra Worden).

The mathematical approach, based on a system of partial derivative differential equations, allows to obtain the vertical distributions of the five picophytoplankton populations as a function of the depth, at different times. The location of the production layer of each group is shown in Fig. 4, where a schematic representation of the mechanism underlying the phytoplankton dynamics is given.

### The model

In this subsection, we study the dynamics of our ecosystem by using a deterministic reaction-diffusion-taxis model [11, 12, 22]. The populations analyzed are distributed along a one-dimensional spatial domain (*z*-direction) of the MAW. In particular, according to biological requirements we assume that the phytoplankton growth is subject to the limiting effect of two external factors, i.e. light intensity and nutrient. By this way it is possible to reproduce the dynamics of the cell concentration of the five populations analyzed, i.e. Synechococcus, Haptophytes, Prochlorococcus HL, Pelagophytes and Prochlorococcus LL, indicated by *b*_1_(*z*,*t*), *b*_2_(*z*,*t*), *b*_3_(*z*,*t*), *b*_4_(*z*,*t*) and *b*_5_(*z*,*t*), respectively. Moreover, the vertical distributions of the nutrient concentration *R*(*z*,*t*) and light intensity *I*(*z*,*t*) are obtained. In our model we also consider the active movement of single microorganisms, the passive movement due to the turbulence, the specific loss rate, and the growth rate of each picophytoplankton population, which plays a crucial role in the overall dynamics of the ecosystem. The limiting effect of light intensity and nutrient concentration, whose values along the water column strongly affect the phytoplankton growth rates [11, 20, 54, 81], is considered by the Monod kinetics [82]. In particular, the gross phytoplankton growth rates per capita are given by $\min\{f_{I_{i}}(I),f_{R_{i}}(R)\}$, where $f_{I_{i}}(I),$ and $f_{R_{i}}(R)$ are obtained by the Michaelis-Menten formulas

$$f_{I_{i}}(I)=r_{i}I/(I+K_{I_{i}}),$$ (1)

$$f_{R_{i}}(R)=r_{i}R/(R+K_{R_{i}}),$$ (2)

Here *r_i_* is the maximum growth rate, $K_{I_{i}}$ and $K_{R_{i}}$ are the half-saturation constants for light intensity and nutrient concentration, respectively, of the *i-th* picophytoplankton group. According to previous studies [14, 16, 17, 22, 83], the constants depend on the metabolism of the specific microorganisms considered. Specifically, the values of $K_{R_{i}}$ and $K_{I_{i}}$ determine, along the water column, the boundaries of the production layer and the position of the maximum of cell concentration for each population.

The specific loss rate of the *i-th* picophytoplankton group, due to respiration, death, and grazing, is given by *m_i_* [11, 12, 22]. In particular, the loss rates have been estimated by using the experimental results collected by other authors [84–87]. Afterwards, we estimated the net per capita growth rates defined as follows

$$G_{i}(z,t)=\min(f_{R_{i}}(R(z,t)),f_{I_{i}}(I(z,t)))-m_{i}.$$ (3)

The passive movement of all phytoplankton groups depends on the turbulence [22], which is modeled by the vertical turbulent diffusivity (*D*(*z*,*t*)). This environmental variable changes as a function of the time, by assuming uniformly larger values *D_U_*(*t*) in the upper layer and uniformly smaller values *D_D_*(*t*) in the deeper layers. The influence of the upper mixed layer on population dynamics has been studied modifying, according to experimental findings, the thickness of UML and the spatial behaviour of vertical turbulent diffusivity depending on the period of the year. The gradual transition from the upper mixed layer to deeper layers has been described in terms of the following generalized Fermi function [22]

$$D(z,t)=D_{D}(t),+\frac{D_{U}(t)-D_{D}(t)}{1+\exp^{(z-Z_{U}(t))/w}},$$ (4)

where *Z_U_*(*t*) is the thickness of the upper mixed layer varying with time, and the parameter *w* is the width of the transient layer.

In our previous works we inserted an advection term which mimics the passive (sinking) movement of the phytoplankton cells [16, 17, 23]. Other authors however assumed that phytoplankton moves along the water column upward (downward), if growing conditions are better above than below (below than above), being motionless if no differences are present between above and below [11]. The correctness of this choice is confirmed by the results of our theoretical analysis. This mechanism in fact contributed to improve the results of our model, allowing to obtain a shape of the DCM in a better agreement with experimental findings. In order to better simulate the behaviour of picophytoplankton populations and provide a more realistic description of our ecosystem, we consider therefore the active movement of the single microorganisms by a taxis term, where the swimming velocity *v_i_* of each population is a function of the gradient of the net growth rate (∂*G_i_*(*z,t*)/∂*z*) [11].

Specifically, as well as in Ref. [11], we fix positive the downward direction of the coordinate *z* (increasing depth). Moreover, the swimming velocity is set positive if the motion occurs downward, an opposite condition respect to that used in Ref. [11]. This choice causes the appearance of the minus sign in the active movement term, instead of the plus sign present in the same term of Ref. [11]. Therefore, in order to reproduce the active movement of the *i-th* picophytoplankton group, we use a step function [11], defined as $v_{i}=+v_{i}^{s}$ if ∂*G_i_*(*z,t*)/∂z > 0, $v_{i}=-v_{i}^{s}$ if ∂*G_i_*(*z,t*)/∂z < 0, and *v_i_* = 0 if ∂*G_i_*(*z,t*)/∂z = 0, where $v_{i}^{s}$ is a constant parameter, whose value (positive) is estimated for each population by using the same criteria adopted by Raven [88].

With these assumptions we obtain the following differential equations for the ecosystem analyzed [11, 12, 14, 22]

$$\frac{\partial b_{i}(z,t)}{\partial t}=b_{i}min(f_{I_{i}}(I)f_{R_{i}}(R))-m_{i}b_{i}+\frac{\partial }{\partial z}\left[D(z,t)\frac{\partial b_{i}(z,t)}{\partial z}\right]-v_{i}\left(\frac{\partial G_{i}(z,t)}{\partial z}\right)\frac{\partial b_{i}(z,t)}{\partial z}.$$ (5)

According to the previous analysis performed for one- and two-population ecosystems [12, 15–17, 22], the cell concentration of the *i-th* picophytoplankton group is subject to the following boundary conditions:

$${\left[D(z,t)\frac{\partial b_{i}}{\partial z}-v_{i}b_{i}\right]|}_{z=0}={\left[D(z,t)\frac{\partial b_{i}}{\partial z}-v_{i}b_{i}\right]|}_{z=z_{b}}=0,$$ (6)

corresponding to the conditions of no-flux both through the water surface (*z* = 0) and interface MAW-LIW (*z* = *z_b_*).

In the model we also take into account a further quantity of nutrient, obtained by the recycling process of dead phytoplankton. Furthermore we recall that, due to the turbulence described by the vertical turbulent diffusivity *D*(*z*,*t*), the nutrient along the water column undergoes a mixing process. The dynamics of the nutrient concentration can be therefore modeled as follows

$$\frac{\partial R(z,t)}{\partial t}=-\sum \frac{b_{i}(z,t)}{Y_{i}}\cdot min(f_{I_{i}}(I),f_{R_{i}}(R))+\frac{\partial }{\partial z}[D(z,t)\frac{\partial R(z,t)}{\partial z}]+\sum \varepsilon_{i}m_{i}\frac{b_{i}(z,t)}{Y_{i}},$$ (7)

where *ε_i_* and 1/*Y_i_* are nutrient recycling coefficient and nutrient content of the *i-th* picophytoplankton group, respectively.

According to the conditions fixed for previous population models [12, 14–17, 22, 23, 89], nutrients are commonly supplied from the bottom. In particular, nutrient concentration at the bottom of the water column, *R*(*z_b_*), is fixed at the average value *R_in_* for the whole period investigated. The boundary conditions for nutrients are therefore modeled by the following equations

$${\frac{\partial R}{\partial z}|}_{z=0}=0,\;R(z_{b})=R_{in}.$$ (8)

The cell concentration *b_i_*(*z,t*), obtained by solving the equations of the system, is then converted in chlorophyll concentration *chla*_i_(*z,t*) for each phytoplankton group. This allows to take into account the shading of the chlorophyll molecules, whose effect is described by the Lamber-Beer’s law [18, 89–91]

$$I(z,t)=I_{in}(t)\exp\left\{-\int_{0}^{z}\left[\sum a_{i}\cdot chla_{i}(Z)+a_{bg}\right]dZ\right\},$$ (9)

with light intensity being characterized by the usual exponential decrease.

In Eq. (9) *a_i_* are the *chl a*-normalized average absorption coefficients of the *i-th* picophytoplankton group, *a_bg_* is the background turbidity, and *I_in_*(*t*) is the incident light intensity at the water surface, changing with the time due to daily and seasonal variations. Equations (5)–(9) describe mathematically the model used to reproduce the spatio-temporal dynamics of the five picophytoplankton populations studied in this work.

### Setting of parameters

In this subsection we describe the procedure to set the values of the environmental and biological parameters used in the model to reproduce the experimental distributions of the total concentration of chlorophyll (see Fig. 3), collected during the oceanographic surveys. In particular, the parameters have been fixed in order to guarantee the coexistence of all five planktonic groups [12, 17, 22, 23, 83], as observed in the sampling site of Tyrrhenian Sea during the period investigated (from 24 November 2006 to 9 June 2007). Moreover, to obtain numerical results in agreement with experimental data, we used periodical functions to simulate seasonal changes.

The numerical values assigned to biological and environmental parameters are shown in Table 1. In particular, the biological parameters have been set to values typical of the five populations investigated: i) maximum specific growth rates according to those measured by other authors [84, 85, 92]; ii) specific loss rates according to experimental results given in previous works [84–87]. Conversely, the swimming velocity and nutrient recycling coefficients have been chosen by following different criteria respect to previous works [16, 17]. Specifically, the magnitudes of swimming velocities of the five picophytoplankton populations are set equal to the values obtained by Raven [88], while nutrient recycling coefficients have been calculated by taking into account the assimilation efficiencies of the planktonic groups estimated by Thingstad [86].

**Table 1:** Parameters used in the model.

**Symbol**	**Interpretation**	**Units**	**Value**
			
*a_bg_*	Background turbidity	m^−1^	0.060
*a*_1_	Average absorption coefficient of Synechococcus	m^2^ mg chl-a ^−1^	0.025
*a*_2_ = *a*_4_	Average absorption coefficient of picoeukaryotes	m^2^ mg chl-a ^−1^	0.012
*a*_3_	Average absorption coefficient of Prochlorococcus HL	m^2^ mg chl-a ^−1^	0.016
*a*_5_	Average absorption coefficient of Prochlorococcus LL	m^2^ mg chl-a ^−1^	0.027
*a*_6_	Average absorption coefficient of phytoplankton > 3*µm*	m^2^ mg chl-a ^−1^	0.020
*r*_1_	Maximum specific growth rate of Synechococcus	h^−1^	0.058
*r*_2_	Maximum specific growth rate of Haptophytes	h^−1^	0.079
*r*_3_	Maximum specific growth rate of Prochlorococcus HL	h^−1^	0.088
*r*_4_	Maximum specific growth rate of Pelagophytes	h^−1^	0.096
*r*_5_	Maximum specific growth rate of Prochlorococcus LL	h^−1^	0.031
$K_{I_{1}}$	Half-saturation constant of light-limited growth of Synechococcus	*µ*mol photons m^−2^ s^−1^	70.00
$K_{I_{2}}$	Half-saturation constant of light-limited growth of Haptophytes	*µ*mol photons m^−2^ s^−1^	90.00
$K_{I_{3}}$	Half-saturation constant of light-limited growth of Prochlorococcus HL	*µ*mol photons m^−2^ s^−1^	40.00
$K_{I_{4}}$	Half-saturation constant of light-limited growth of Pelagophytes	*µ*mol photons m^−2^ s^−1^	35.00
$K_{I_{5}}$	Half-saturation constant of light-limited growth of Prochlorococcus LL	*µ*mol photons m^−2^ s^−1^	6.00
$K_{R_{1}}$	Half-saturation constant of nutrient-limited growth of Synechococcus	mmol phosphorus m^−3^	0.00001
$K_{R_{2}}$	Half-saturation constant of nutrient-limited growth of Haptophytes	mmol phosphorus m^−3^	0.00004
$K_{R_{3}}=K_{R_{5}}$	Half-saturation constant of nutrient-limited growth of Prochlorococcus HL	mmol phosphorus m^−3^	0.00200
$K_{R_{4}}$	Half-saturation constant of nutrient-limited growth of Pelagophytes	mmol phosphorus m^−3^	0.01190
*m*_1_	Specific loss rate of Synechococcus	h^−1^	0.014
*m*_2_ = *m*_4_	Specific loss rate of picoeukaryotes	h^−1^	0.010
*m*_3_ = *m*_5_	Specific loss rate of Prochlorococcus	h^−1^	0.011
1/*Y*_1_	Nutrient content of Synechococcus	mmol phosphorus cell^−1^	2.86 × 10^−14^
1/*Y*_2_ = 1/*Y*_4_	Nutrient content of picoeukaryotes	mmol phosphorus cell^−1^	2.00 × 10^−12^
1/*Y*_3_ = 1/*Y*_5_	Nutrient content of Prochlorococcus	mmol phosphorus cell^−1^	1.33 × 10^−13^
*c*_1_	Chl-a cellular content of Synechococcus	fg chl-a cell^−1^	2.00
*c*_2_ = *c*_4_	Chl-a cellular content of picoeukaryotes (as a function of depth)	fg chl-a cell^−1^	10.00 − 660.00
*c*_3_ = *c*_5_	Dvchl-a cellular content of Prochlorococcus (as a function of depth)	fg Dvchl-a cell^−1^	0.25 − 2.20
*ε*_1_	Nutrient recycling coefficient of Synechococcus	dimensionless	0.51
*ε*_2_ = *ε*_4_	Nutrient recycling coefficient of picoeukaryotes	dimensionless	0.52
*ε*_3_ = *ε*_5_	Nutrient recycling coefficient of Prochlorococcus	dimensionless	0.52
$v_{1}^{s}$	Magnitude of swimming velocity of Synechococcus	m h^−1^	0.000088
$v_{2}^{s}=v_{4}^{s}$	Magnitude of swimming velocity of picoeukaryotes	m h^−1^	0.000098
$v_{3}^{s}=v_{5}^{s}$	Magnitude of swimming velocity of Prochlorococcus	m h^−1^	0.000039
*z_b_*	Depth of the water column	m	200
*R_in_*	Nutrient concentration at *z_b_*	mmol phosphorus m^−3^	0.204

The values of the biological and environmental parameters are those typical of five picophytoplankton populations that coexist in the Tyrrhenian Sea during the whole year (Refs. [16–18, 69, 70, 74, 78, 84, 85, 85–88, 92, 93]).

The half-saturation constants, $K_{R_{i}}$ and $K_{I_{i}}$, for the five populations are set so that the production layers and peaks of cell concentration are located at depths compatible with measured values. In particular, the values of half-saturation constants $K_{I_{i}}$, chosen according to previous experimental results [78, 93], result to be small for those populations, such as Pelagophytes and Prochlorococcus LL, which are better adapted to low light intensities. On the other side, the half-saturation constants $K_{R_{i}}$ are set at low values for those populations, such as Synechococcus and Haptophytes, which are better adapted to low nutrient concentrations. As a consequence, the peaks of abundance of Pelagophytes and Prochlorococcus LL are localized, along the water column, deeper than those of the Synechococcus and Haptophytes. All half-saturation constants are set to the same values for the whole period investigated (see Table 1). Finally we note that the values chosen for $K_{R_{i}}$ ensure, during the whole year, phosphorus concentrations sufficient for the survivance of the picophytoplankton groups located in the shallower layers [14, 12].

The nutrient contents of the picophytoplankton groups, 1/*Y_i_*, are fixed to the same values for all sampling periods (see Table 1). The values of these parameters have been estimated for Synechococcus and Pelagophytes by using previous works [93, 94], while no data are available for the other picophytoplankton groups. Therefore, in order to obtain cell concentrations in agreement with the experimental findings, we set the nutrient contents of Haptophytes and Prochlorococcus (both ecotypes) in such a way to respect, for the ratios of the average concentrations of different populations, the values experimentally observed in the Strait of Sicily [70, 74].

The *chl a*-normalized average absorption coefficients have been estimated by using the light absorption spectra obtained by other authors from analyses on phytoplankton cultures [18, 78, 95]. Specifically, the values used in the model are in agreement with the absorption coefficients measured by Brunet et al. in Gulf of Naples [96].

The environmental parameters have been chosen to reproduce the marine ecosystem of the Tyrrhenian Sea during the whole year. The water column depth used in the model is fixed equal to that estimated for the MAW (200 m). The vertical turbulent diffusivity in the deep layers *D_D_*(*t*) changes as a function of the time [68], taking on values typical of weakly mixed waters (*D_D_*(*t*) ≤ 6.0 cm^2^ s^-1^, in all seasons). In particular, in order to better reproduce the experimental profiles, we fixed the vertical turbulent diffusivity in the deeper layers (see Table 2), using theoretical values in agreement with previous works [17, 22, 23, 68]. Conversely, in the upper mixed layer the diffusivity *D_U_*(*t*) takes on, during the year, different values which can be estimated by exploiting methods adopted by other authors in marine ecosystems [63–67, 97]. Specifically, to obtain *D_U_* for each sampling period, we used the expression of Denman and Gargett [64]

$$D_{U}=0.25\epsilon N^{-2},$$ (10)

where *ϵ* and *N* are the turbulent kinetic energy dissipation rate in UML and the buoyancy frequency, respectively.

**Table 2:** Monthly average values of vertical turbulent diffusivity in deeper layers (*D_D_*) and in upper mixed layer (*D_U_*), thickness of the upper mixing layer (*Z_U_*) and incident light intensity (*I*_*in*_).

**Month**	*D_D_*(m^2^ h^−1^)	*D_U_*(m^2^ h^−1^)	*Z_U_*(m)	*I_in_*(*µ*mol ph. m^−2^s^−1^)
				
January	1.059	5.503	70.311	433.058
February	0.903	9.277	58.849	623.864
March	0.699	6.722	37.308	904.648
April	0.513	4.169	16.490	1154.665
May	0.684	3.148	17.250	1369.813
June	0.889	2.652	22.260	1518.738
July	0.900	2.198	20.886	1526.101
August	0.947	1.828	19.925	1336.145
September	1.357	2.140	25.247	1020.497
October	1.845	2.597	31.944	726.194
November	2.056	2.462	44.784	463.077
December	1.601	3.032	59.981	364.566

The turbulent kinetic energy dissipation rate is calculated by using the following expression of Turner [64, 98]

$$\epsilon =u_{*}^{3}/(k\cdot Z_{U}),$$ (11)

where u_*_ is the turbulent friction velocity in the water surface, *k* = 0.4 is the von Karman constant, and *Z_U_* is the thickness of the upper mixing layer. The turbulent friction velocity is estimated by the monthly average wind speed measured at 10 m above the sea surface, available on the web site of the CISL Research Data Archive (http://rda.ucar.edu/datasets/ds540.1/). Moreover, according to previous works [62, 64], we consider the thickness of the upper mixing layer *Z_U_* equals to the minimum between the turbulent Ekman layer thickness *Z_e_* and the depth, *Z_t_*, of the thermocline. The former is calculated using the turbulent friction velocity in the water surface and the Coriolis parameter estimated in the site analyzed, the latter is obtained by the vertical profile of temperature acquired in situ for each sampling period.

The buoyancy frequency *N* can be calculated directly by the vertical profile of water density as follows

$$N^{2}=(g/\rho_{w})\cdot \frac{\partial \rho (z)}{\partial z},$$ (12)

where *g* is the gravity acceleration, ρ*_w_* is the density of the sea water, and $\frac{\partial \rho (z)}{\partial z}$ is the vertical density gradient between the UML and the deeper layers of the water column.

**Figure 5:**
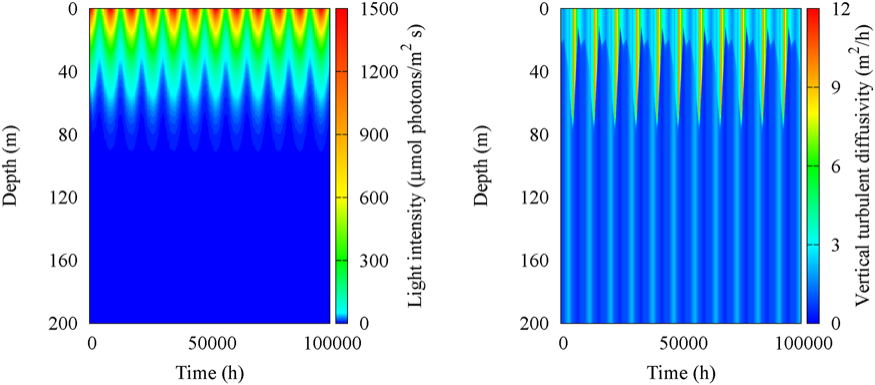
Spatio-temporal behaviour of vertical turbulent diffusivity (left panel) and light intensity (right panel) simulated for the sampling site (39° 30′.00 N, 13°30′.00 E). The values of the parameters are those of Table 2.

By this way, the vertical turbulent diffusivity *D_U_* and thickness *Z_U_* of the UML were estimated by using the experimental profiles of temperature and density, collected in the sampling site (39°30.00′N,13°30.00′E) during the oceanographic surveys (see subsection Environmental data). The vertical profile of diffusivity, along the whole water column, was obtained for each sampling period by using Eq. (4). The spatio-temporal behaviour of the vertical turbulent diffusivity has been reproduced by interpolating the theoretical results obtained for all periods of the year investigated (see Fig. 5a). Specifically, the daily dynamics of diffusivity was simulated by using a step function. The monthly average values of the vertical turbulent diffusivity *D_U_* and thickness *Z_U_* of the UML are given in Table 2. The light intensity at the water surface, *I_in_*(*t*), was estimated for all days of the year, taking into account the daily average values available on the NASA web site (http://eosweb.larc.nasa.gov/sse/RETScreen/). The spatio-temporal behaviour of the incident light intensity is shown in Fig. 5b, while the monthly average values are given in Table 2. Finally, nutrient concentrations at depth *z_b_* were fixed at the average value of phosphorus concentration (*R_in_* = 0.204 mmol m^-3^) obtained by analyzing the bottle samples collected in the site investigated during the oceanographic surveys.

## Results

The theoretical distributions of cell concentration for the five picophytoplankton populations were obtained by solving numerically Eqs. (5)–(9). In particular, we used a numerical method, whose computer implementation consists in a C++ program, based on an explicit finite difference scheme with centered-in-space differencing for the diffusion term and upwind differencing for the taxis term. The increment of the spatial variable and the time step are set at 0.5 m and 0.05 h, respectively. Specifically, these values are chosen such as to obtain the stability conditions for both differencing terms. Moreover, on the basis of the stability analysis performed according to previous works [99–104], the convergence of the whole finite difference equations is guaranteed [100, 103, 104].

As initial conditions, we assumed for each picophytoplankton group a small cell concentration uniformly distributed along the water column in agreement with Ryabov et al. [22], while the nutrient concentration is fixed equal to zero from the water surface to the thermocline, with a linear increase below this point up to the interface MAW-LIW.

**Figure 6:**
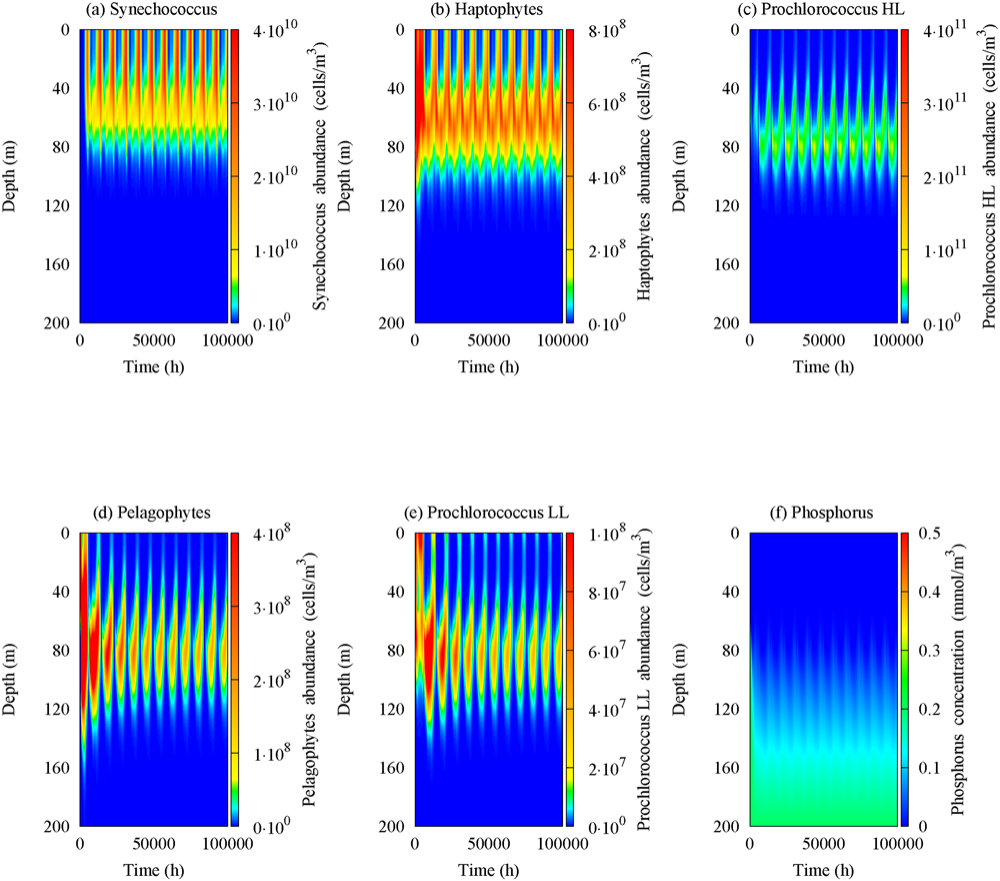
Spatio-temporal behaviour of the five picophytoplankton groups and phosphorus concentrations simulated by the model. The contour maps show the cell concentrations of (a) Synechococcus, (b) Haptophytes, (c) Prochlorococcus HL, (d) Pelagophytes, (e) Prochlorococcus LL and (f) nutrient. The values of the parameters used in the model are those shown in Tables 1 and 2.

The equation system (5)–(9) is integrated over a period of approximately eleven years. The solution, after nine years, reaches a stationary regime characterized by a periodical behaviour. Therefore, setting as an integration time *t_max_* = 10^5^ *h*, we obtain steady seasonal driven oscillations of picophytoplankton abundances and nutrient concentration corresponding to all periods experimentally investigated (four data sets at different times within one year (see Fig. 3)). The numerical results are shown in Fig. 6. Here, it is possible to observe the presence of the chlorophyll peak for Haptophytes, Prochlorococcus HL and Pelagophytes in intermediate layers of MAW, in correspondence of the experimental DCM (see Fig. 3), during the whole year. In particular, the cell concentrations of Prochlorococcus HL and Pelagophytes decrease during late fall and winter, with a strong enhancement in spring and summer seasons. These theoretical results are in agreement, not only with our field observations, but also with experimental data previously obtained in the Strait of Sicily and the Bay of Naples [59, 70, 74]. Moreover, a Synechococcus abundance peak is always observed close to the surface water in correspondence of the upper mixed layer. Specifically, the cell concentration of Synechococcus increases during the late fall up to reach a maximum value in winter season, while decreases between early spring and late summer. The peak of Prochlorococcus LL cell concentration is localized in deeper layers, where it takes on very low values during the whole year (see Fig. 6), according to experimental data collected in Tyrrhenian Sea during the last years.

In general, the numerical results show that the high cell concentration of Synechococcus and Haptophytes, observed during late fall and winter, is accompanied by a decrease in the abundance of the other groups localized in deeper layers [14, 83]. Vice versa, Prochlorococcus and Pelagophytes dominate the water column in spring and summer seasons, when their presence in the deeper layers inhibits the nutrient uptake for the groups localized in UML. Also in this case, the theoretical results are in agreement with the experimental data acquired during the period investigated. Finally, we note that the nutrient concentration show the typical behaviour experimentally observed in bottle samples acquired during the oceanographic surveys. Specifically, the phosphorus concentration remains almost constant close to the interface MAW-LIW in all seasons.

**Figure 7:**
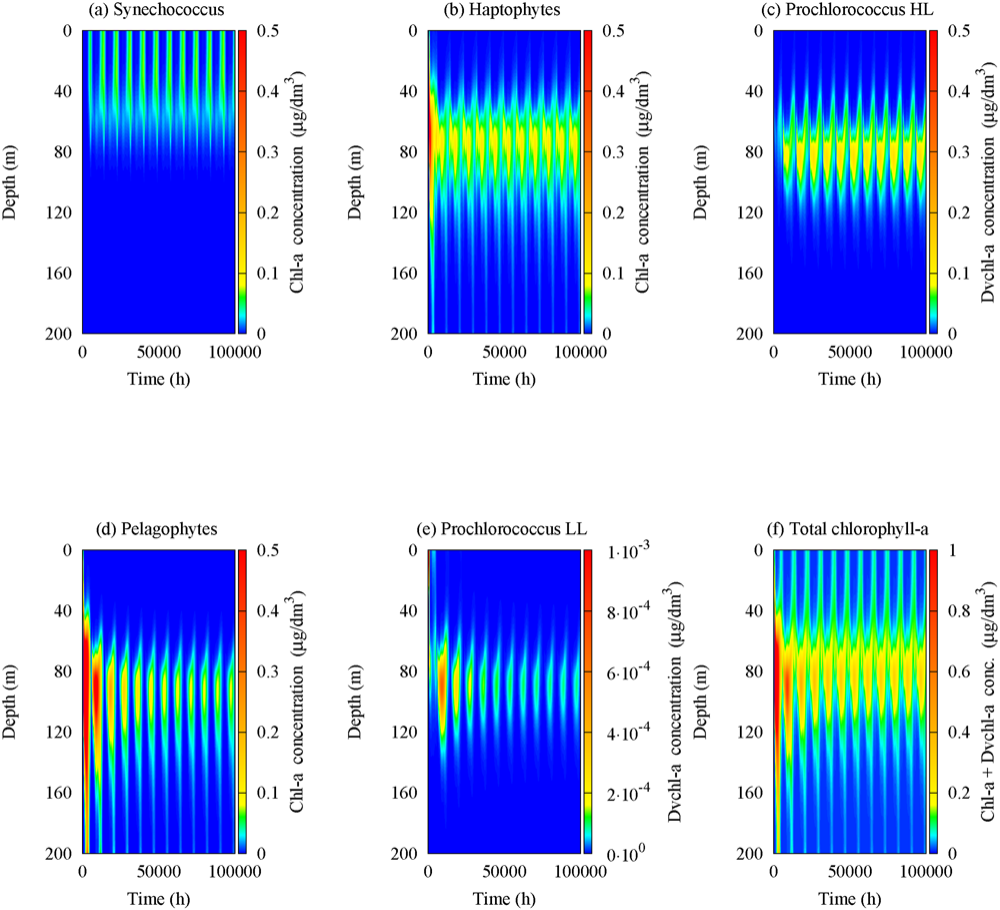
Spatio-temporal behaviour of *chl a* and *Dvchl a* concentrations. Contour maps show the content of chlorophyll for (a) Synechococcus, (b) Haptophytes, (c) Prochlorococcus HL, (d) Pelagophytes, (e) Prochlorococcus LL and (f) all phytoplankton groups in the sampling site (39° 30′.00 N, 13°30′.00 E). The values of the parameters used in the model are those shown in Tables 1 and 2.

We recall that the experimental profiles for *chl a* concentrations are expressed in *μ*g/dm^3^ (see Fig. 3). As a consequence, in order to compare the theoretical results with the experimental findings, the numerical cell concentrations of the five populations (expressed in cells/m^3^) were converted into *chl a* and *Dvchl a* concentrations, setting the cellular content of Synechococcus equal to 2 fg *chl a* cell^-1^ [69], and using the curves of mean vertical profile for the other groups [70, 74]. The theoretical profiles, resulting by the conversion performed for each picophytoplankton group, are shown in Fig. 7.

**Figure 8:**
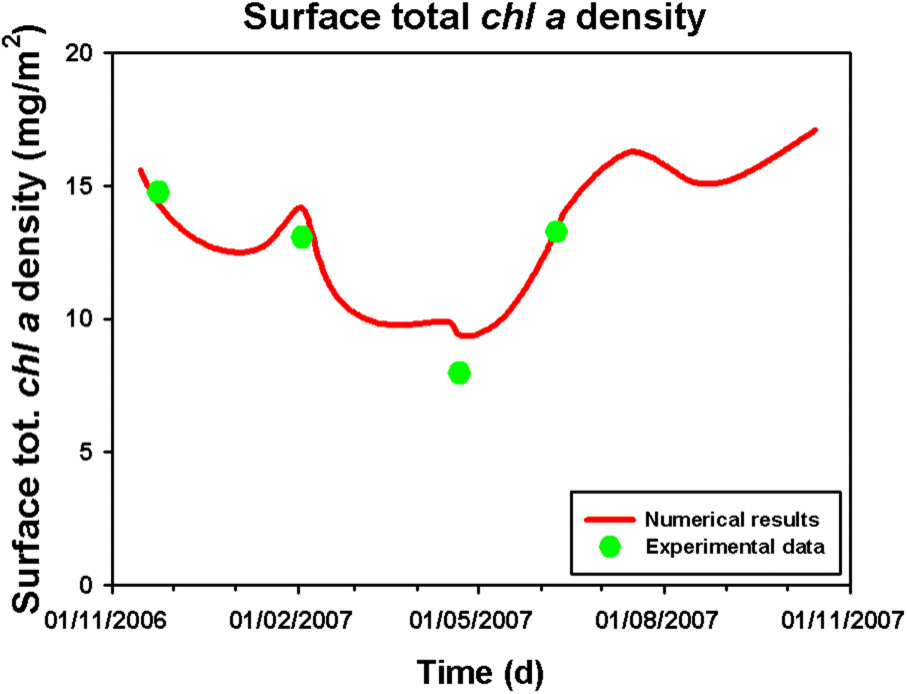
Surface total *chl a* density as a function of time. Red line shows, for a yearly cycle with a time resolution of one month, the surface total chl-a density obtained by integrating the theoretical profiles of the total *chl a* and *Dvchl a* concentration over the MAW (from the surface down to 200 m). The experimental data (green points) for the site analyzed (39° 30′.00 N, 13°30′.00 E) are those acquired in correspondence of the four sampling periods (VECTOR-TM1, 24 November 2006; VECTOR-TM2, 3 February 2007; VECTOR-TM3, 22 April 2007; VECTOR-TM4, 9 June 2007).

It is worth to recall that the structure of the *chlorophyll a* molecule is almost identical to that of *divinyl chlorophyll a*, therefore we can sum their concentrations (without introducing significant errors) to obtain the theoretical equilibrium profiles for the total *chl a* and *Dvchl a* concentration. Moreover, we performed the HPLC analysis on the content of the bottle samples collected in the same site of the Tyrrhenian Sea during different periods of the year, observing the presence of diatoms, cryptophytes and dinophytes in traces. As a consequence their contribution to the total *chlorophyll a* concentration can be neglected. Conversely, it has been estimated that the fraction of nano- and micro-phytoplankton (> 3*μm*) accounts about for 20% of the total quantity of *chl a* and *Dvchl a*. On the basis of the bottle samples analyzed, the total amount of *chl a* and *Dvchl a* can be considered uniformly distributed in the MAW. Thus, following the same procedure as in previous works [15–17, 89], we calculated the mean chlorophyll concentration of nano- and micro-phytoplankton and divided it by the length of the water column, obtaining Δ*b*_(*Dv*)*chl a*_, which is a constant value of chlorophyll concentration along the whole water column, due to other phytoplankton groups present in the site investigated [16, 17]. In particular the mean chlorophyll concentration of nano- and micro-phytoplankton is obtained averaging on both space (the first 200 meters of the water column) and time (the whole period during which the four samplings were performed, i.e. from 24 November 2006 to 9 June 2007). Therefore, we added the numerical concentrations with Δ*b*_(*Dv*)*chl a*_ and obtained, for the total *chl a* and *Dvchl a* concentration, the spatio-temporal behaviour shown in Fig. 7. Here, we note that the DCM is present during the whole year, even if a strong increase of chlorophyll concentration is observed in the surface layer during late fall and winter, when the total average *chl a* and *Dvchl a* concentration reaches a maximum value equal to 0.10 *μ*g/l. These results are in agreement with the experimental profiles collected during the oceanographic surveys considered in this work. In particular, they indicate that the upwelling of nutrients along the water column, due to an increase of vertical turbulent diffusivity in UML during late fall and winter, supports the growth of Synechococcus and Haptophytes, determining an increase of the total *chl a* and *Dvchl a* concentration in the shallower layers. Conversely, the numerical results show in the same period a strong decrease of the total *chl a* and *Dvchl a* concentration in the intermediate and deeper layers of the water column, due to a reduced mixing below the thermocline. This causes a decrease of surface total *chl a* concentration in the MAW (from the surface down to 200 m) up to reach the minimum value in April (see Fig. 8).

**Figure 9:**
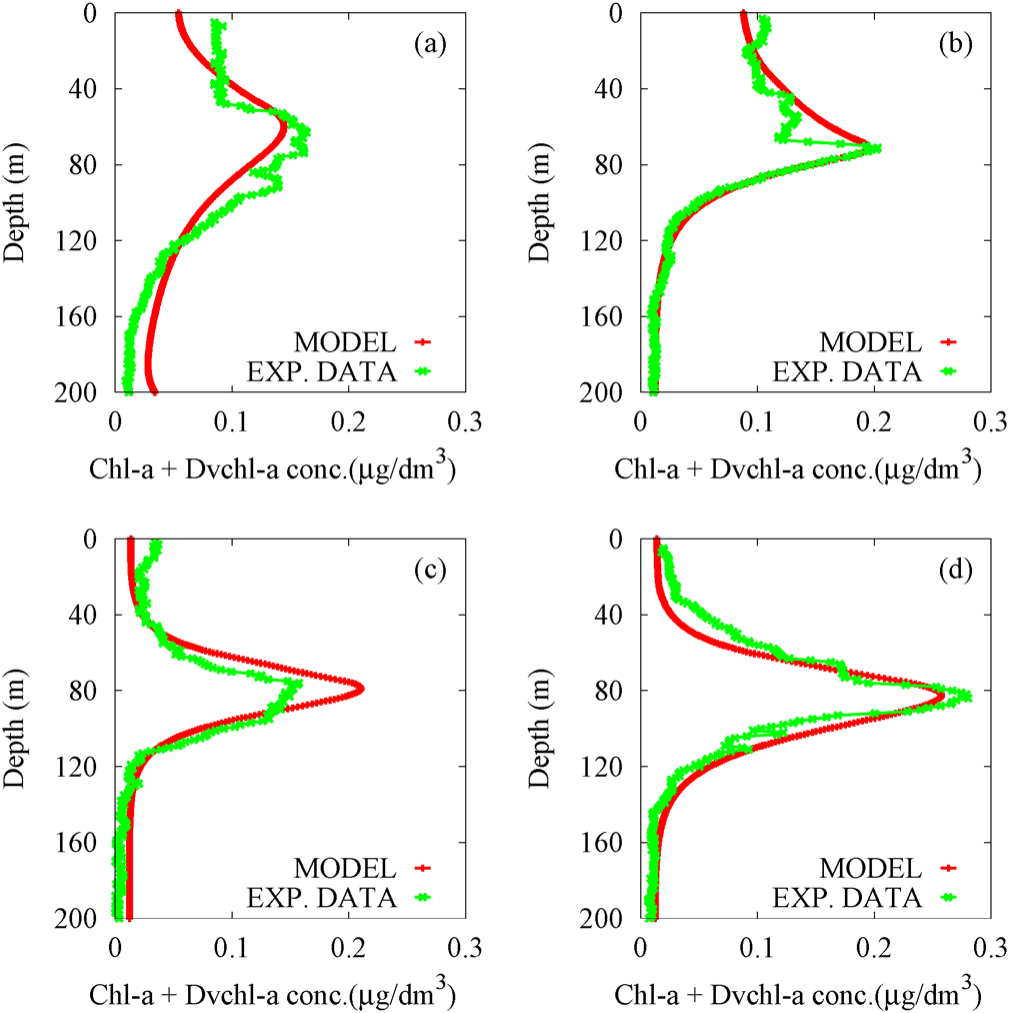
Theoretical distributions (red line) and experimental profiles (green line) of the total *chl a* and *Dvchl a* concentration. The numerical results, obtained by the five-population model and given as a function of the depth, are compared with the experimental data collected in the sampling site (39° 30′.00N, 13°30′.00E), during the oceanographic surveys: VECTOR-TM1, 24 November 2006 (panel a); VECTOR-TM2, 3 February 2007 (panel b); VECTOR-TM3, 22 April 2007 (panel c); VECTOR-TM4, 9 June 2007 (panel d).

Finally, we compared the experimental profiles acquired during oceanographic surveys (VECTOR-TM1, 24 November 2006; VECTOR-TM2, 3 February 2007; VECTOR-TM3, 22 April 2007; VECTOR-TM4, 9 June 2007) with the theoretical distributions extracted by contour maps (see Fig. 7) in correspondence of the same sampling periods. The results, shown in Fig. 9, indicate the presence of a good agreement between experimental data (green line) and numerical results (red line) in all seasons. In particular, by performing the goodness-of-fit test *χ* ^2^, we obtained the best reduced chi-square, ${\tilde{\chi }}^{2}=0.0014$, in winter (3 February 2007), when the vertical turbulent diffusivity reaches the maximum value in upper mixed layer. In other sampling periods, the results of *χ* ^2^ test (see Table 3) indicate a worse agreement between theoretical and experimental profiles, even if the reduced chi-square ${\tilde{\chi }}^{2}$ takes on values comparable with those obtained in previous works, where two-population models were used to study the phytoplankton dynamics in summer season [17, 23, 89]. Specifically, the numerical results in the upper mixed layer show a good agreement with the experimental data in all sampling periods except for autumn (see Fig. 9). Vice versa, in deeper layers, the *χ* ^2^ test indicates that the magnitude of the total *chl a* and *Dvchl a* concentration close to the DCM is underestimated in autumn (24 November 2006) and in late spring (9 June 2007), while it is overestimated in early spring (22 April 2007).

**Table 3:** Results of *χ* ^2^ and reduced chi-square (${\tilde{\chi }}^{2}$) goodness-of-fit test for the site investigated in correspondence of the four sampling periods.

**Sampling day**	**χ^2^**	${\tilde{\chi }}^{2}$
		
24 November 2006	1.17	0.0060
3 February 2007	0.28	0.0014
22 April 2007	1.32	0.0068
9 June 2007	1.31	0.0067

The number of samples, used for the test and distanced of 1 m, is n = 200 corresponding to consider from the surface the first 200 m of depth.

**Figure 10:**
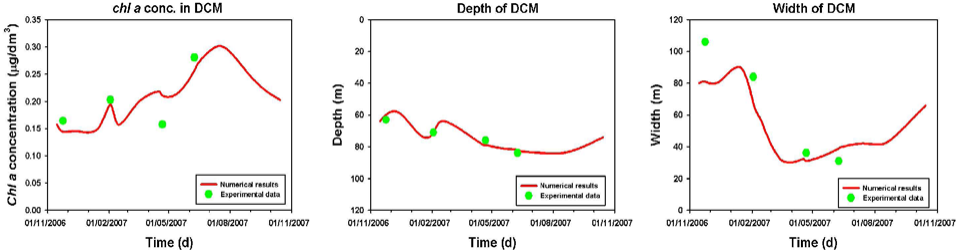
Magnitude, depth, and width of the DCM, as a function of time. Red lines indicate theoretical results obtained for a yearly cycle with a time resolution of one month. Green points indicate experimental data acquired in the site analyzed (39° 30′.00 N, 13°30′.00 E) in correspondence of the four sampling periods (VECTOR-TM1, 24 November 2006; VECTOR-TM2, 3 February 2007; VECTOR-TM3, 22 April 2007; VECTOR-TM4, 9 June 2007).

**Figure 11:**
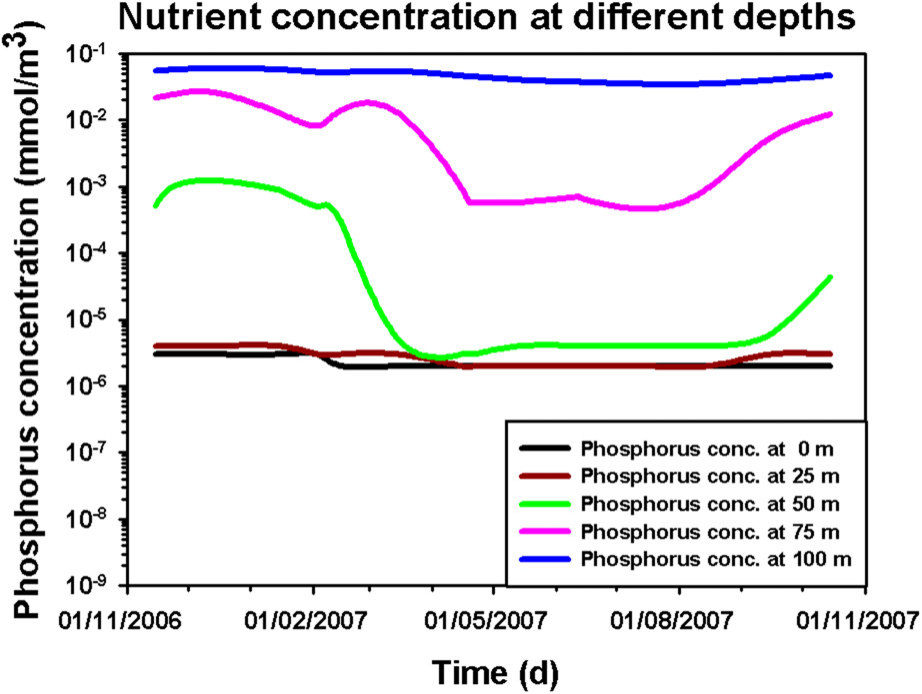
Phosphorus concentration at different depths as a function of time. The figure shows, for a yearly cycle, the theoretical results of phosphorus concentration at 0 m (black line), 25 m (brown line), 50 m (green line), 75 m (pink line) and 100 m (blue line) of depth.

In order to better analyze the effects of seasonal changes on the phytoplankton dynamics, we studied the behaviour of magnitude, depth and width of the DCM as a function of the time (see Fig. 10). The numerical results show that the magnitude of total *chl a* and *Dvchl a* concentration increases in DCM between autumn and winter (see Fig. 10), due to the enhancement of the vertical turbulent diffusivity in UML. During the spring, the reduced mixing in deeper layers generates an increase of magnitude of the DCM, which reaches a maximum value in June. Afterwards, the persistent weak mixing leads to a decrease of nutrient concentration along the whole water column (see Fig. 11), which causes the total *chl a* and *Dvchl a* concentration to diminish between summer and early autumn in UML. The numerical results are in agreement with experimental findings during the whole period investigated except for the sampling of April.

The analysis of the theoretical results also shows an increase of the depth of the DCM between late fall and late spring. Specifically, the maximum value of total *chl a* and *Dvchl a* concentration is observed at 82 m of depth in June. This behaviour is due to an enhancement of the thickness of the upper mixing layer in autumn, followed by a strong reduction of the mixing in the surface waters between winter and early spring. Vice versa, during the same period, the width of the DCM decreases up to reach a minimum value of 31 m in spring, when the limited mixing in the deeper layers generates a strong stratification of the water masse, which persists in summer and early fall. The numerical results give values of depth and width of the DCM in a very good agreement with the experimental findings for all sampling periods.

We note, however, that the small discrepancies between theoretical and experimental distributions can be explained considering three different elements: i) the difficulty to find the correct value for the vertical turbulent diffusivity in the UML during autumn, when the turbulent kinetic energy dissipation rate can increase in unpredictable way and the methods used in this study can give values of *D_U_* lower than those postulated for the real marine ecosystems; ii) the lack of experimental data on the fluctuating velocity components, required to estimate the vertical turbulent diffusivity in deeper layers (*D_D_*) during the year; iii) the half-saturation constants of the nutrient-limited growth are assumed constant, for the whole year, without taking in account their periodical fluctuations due to the seasonal variations of the turbulent kinetic energy dissipation rate. Therefore, in order to better reproduce the vertical profiles of the total *chl a* and *Dvchl a* concentration, we would need an accurate estimate of some physical variables, i.e. the turbulent kinetic energy dissipation rate and fluctuating velocity components, whose experimental values are not available. Finally we note that an alternative theoretical approach could be based on the Droop model, which permits to derive the half-saturation constants of the nutrient-limited growth from mechanisms of nutrient uptake, allowing to reproduce their dynamical changes [105].

In conclusion, the five-population model devised is able to mimics the spatio-temporal behaviour of the picophytoplankton groups when regarding the resulting conversion into total *chlorophyll a* vertical profile. On the other hand, the study indicates that a further improvement is possible, in view of obtaining theoretical prediction in a still better agreement with field observations.

## Discussion

In this work we presented a study on spatio-temporal dynamics of phytoplankton in a real marine ecosystem. The work included the analysis of vertical distributions of chlorophyll concentrations obtained from data sampled, at different times of the year, in a site of the Tyrrhenian Sea, along the south-western Italian coast. The data analysis showed that the properties of chlorophyll profiles depend on the sampling period, evidencing the presence of a strong correlation with the seasonal changes of environmental variables. Moreover, in order to provide a theoretical description of the spatio-temporal behaviour of chlorophyll concentration, we introduced a one-dimensional model [15–17, 89] for the seasonal distribution of phytoplankton groups in a marine ecosystem. In particular, we focused on five picophytoplankton populations, belonging to two different domains, i.e. picoeukaryotes and picoprokaryotes, which account about for 80% of the average value of total chlorophyll in Tyrrhenian Sea and represent the whole smaller size fraction (less than 3 *μm*) of the phytoplankton biomass. The theoretical analysis allowed to get the cell concentration distributions along the water column, in a fixed marine site, during a period of eleven years, providing a clear idea of the spatio-temporal dynamics of picophytoplankton concentration. Specifically, we analyzed the theoretical profiles of phytoplankton abundances, obtained at four different times, corresponding to the periods of the year during which the experimental chlorophyll data were sampled. We devised our model, taking into account the biological and environmental properties of the marine ecosystem investigated. Indeed the site analyzed is characterized by oligotrophic waters, typical of Tyrrhenian Sea. Here, the chlorophyll concentration decreased during the last years due to a reduced abundance of nutrients in the upper mixed layer, generated by a more stable stratification of the Mediterranean basin [1, 106, 107]. Therefore the surface mixed layer is depleted in nutrients, and subsurface maxima of chlorophyll concentration are often found. Such deep chlorophyll maxima (DCMs) are permanent features in many tropical and subtropical oceans [16, 89, 108–112]. Moreover, seasonal DCMs commonly develop in temperate regions [16, 89, 111, 113, 114] and even in polar oceans [115], when the surface layer are depleted in nutrients at the beginning of the summer season. In the southern Tyrrhenian Sea, blooms are not enough to suggest an increase in phytoplankton abundance associated to the upwelling process during the winter, even if an increase of chlorophyll concentration is observed in the upper mixed layer during late fall and early winter [53, 116].

Our theoretical analysis, based on a one-dimensional reaction-diffusion-taxis model, is devoted to understand the deep mechanisms which govern the phytoplankton dynamics. We therefore outlined a general approach to predict the spatio-temporal behaviour of chlorophyll distributions in marine ecosystems. For this purpose we took into account the biological and environmental characteristics of a specific ecosystem, using parameter values biologically and physically meaningful, which allow to reproduce the chlorophyll distributions measured during oceanographic surveys. We therefore reproduced the spatio-temporal dynamics of the total *chl a* and *Dvchl a* concentration by taking into account the effects of seasonal variations of environmental variables. In particular, we solved the equations of our model, considering the spatio-temporal behaviour of the vertical turbulent diffusivity, *D*(*z,t*), which is simulated by using the methods adopted in previous works [22, 61, 64]. Specifically, the vertical turbulent diffusivity in the upper mixed layer, *D_U_*, was estimated by taking into account the seasonal changes occurring in the following quantities: i) average wind speed above the sea surface; ii) depth of the thermocline; iii) vertical profile of salinity [62–65, 67, 98]. On the other side, the vertical turbulent diffusivity in the deeper layers, *D_D_*, was fixed such as to mimic the hydrodynamic conditions of the marine ecosystem investigated in all periods considered [71]. In general, the values obtained for the vertical turbulent diffusivity, along the water column, ensure the absence of intrinsic oscillations of the picophytoplankton populations in all seasons, maintaining the system far from the chaos [13, 22]. The other environmental variables were estimated by analyzing the experimental data acquired during the four sampling periods. Finally, the biological parameters were fixed according to previous experimental findings [84–87, 93, 94].

By solving the equations of our model, we obtained the spatio-temporal behaviour of the abundance of each phytoplankton population, expressed in number of cells/*m^3^*. We note that the parameter setting allowed to obtain the coexistence of five groups, i.e. Synechococcus, Haptophytes, Prochlorococcus HL, Pelagophytes and Prochlorococcus LL, along the whole water column. This is in agreement with the experimental findings which reveal the presence of the same five picophytoplankton populations in the bottle samples collected in the site (Tyrrhenian Sea) analyzed in this work. Moreover, no bistability phenomenon was observed, varying the initial conditions for phytoplankton abundance and nutrient.

In order to compare theoretical and experimental distributions, the numerical results giving the phytoplankton abundances were converted in *chl a* and *Dvchl a* concentrations, obtaining the theoretical chlorophyll distributions [69, 70]. These profiles, according to the experimental ones, resulted to be strongly dependent on the period considered. In particular, from a qualitative point of view, the DCM (both observed and predicted) presented a width of few meters during the spring and summer seasons, when the behaviour of the vertical turbulent diffusivity guarantees the condition of weakly mixed waters along the whole water column. Vice versa, during late autumn, both experimental and theoretical chlorophyll profiles show the presence of a broadening in the DCM, corresponding to an increase of the vertical turbulent diffusivity in the deeper layers. Finally, during the winter, the theoretical profiles were characterized by high values of the total *chl a* and *Dvchl a* concentration above the thermocline, which can be related to the strong enhancement of the mixing (modeled by high values of the vertical turbulent diffusivity) in the shallower layers. In general, the numerical results showed that the thickness of DCM was comparable with that observed in the experimental data for all sampling periods.

From a quantitative point of view, the *χ* ^2^ goodness-of-fit test indicated the presence of a good agreement between experimental and theoretical findings during the whole period analyzed. In particular, the statistical test showed that the best reduced chi-square is obtained in winter season, when the upwelling of nutrient occurs, due to an increase of vertical turbulent diffusivity in the upper mixed layer. Results not shown here indicated that the worst value of *χ* ^2^ test is obtained by setting the diffusivity in the UML at a constant value during all the year, that is neglecting seasonal variations. Moreover, we studied the spatio-temporal behaviour of only two (Prochlorococcus HL and Pelagophytes) or three picophytoplankton populations (Prochlorococcus HL, Pelagophytes, and Prochlorococcus LL) localized in intermediate layers of the water column. In these cases, the results of of *χ* ^2^ test showed a worse agreement with experimental data respect to the five-populations model. Specifically, theoretical results (here not reported) indicated that the high concentration of *chlorophyll a* close to the water surface, during late fall and winter, can be explained only with the presence of Synechococcus and Haptophytes. On the other side, the correct theoretical chlorophyll-a distributions can not be obtained in springer and summer, without considering Prochlorococcus and Pelagophytes. It is interesting to note that, within the Prochlorococcus genus, the ecotype LL gives a quite small contribution to the *Dvchl a* concentration. An effective theoretical study could be therefore performed also using a four-population model. Anyway, the five-population model allows: i) to consider the main populations present in the water column; ii) to obtain from the model the prevalence in abundance of Prochlorococcus HL on Prochlorococcus LL, according to field observations in Tyrrhenian Sea.

This analysis clearly indicates that the model is able to reproduce experimental data, if the time evolution of the diffusivity and, in general, of environmental parameters is included in the equations. Moreover, we note that this model represents an effective predictive tool, able to reproduce the chlorophyll distributions measured during four different oceanographic surveys. We note that a partial mismatch between predicted and observed chlorophyll profiles appears in the deeper layers during late fall and early spring. This suggests that a better modeling needs a deeper knowledge of environmental parameters such as: i) the values of the vertical turbulent diffusivity in the deeper layers; ii) the dependence of the half-saturation constants, $K_{R_{i}}$, on seasonal variations of the turbulent kinetic energy dissipation rate; iii) the displacements of the phytoplankton biomass along the water column, due to the internal wave motion and Langmuir circulation [64, 67]; iv) the inflow and outflow of nutrients and chlorophyll coming from adjacent sites, if the marine ecosystem is considered as a habitat with three dimensions.

Moreover, a deeper knowledge of the biological mechanism through which the phytoplankton cells absorb nutrients would be necessary. In particular, we could consider the idea that the growth rate of picophytoplankton groups depends on intra-cellular nutrient concentration or cell quota (Droop model), instead of the extra-cellular available nutrient [117]. In this case, the uptake nutrient term of Eq. (7) would be replaced by a new term that takes into account both the extra-cellular nutrient concentration and the cell quota. In general, the theoretical results obtained by Droop model are in agreement with the results of experiments performed with phytoplankton cells in the presence of phosphorus and other nutrients. In particular, the experimental findings have shown that the growth rate is more closely related to the intra-cellular nutrient concentration than to the external one [117]. The Droop model however does not allow to use the Liebig’s minimum law, which permits to take into account the contemporary presence of two limiting factors, i.e. light intensity and nutrient concentration, which plays a crucial role in the overall dynamics of phytoplankton populations. All biological and environmental factors contribute to determine the spatio-temporal dynamics of phytoplankton distributions and, therefore, of chlorophyll profiles. In particular, the internal wave motion generates an oscillating movement of the phytoplankton biomass around the thermocline with a maximum amplitude of 15 m near the continental shelf of the oceans, while the Langmuir circulation determines a circular motion of planktonic groups in the first 5 m of the water column. These phenomena are usually neglected because of their limited impact on the overall phytoplankton dynamics [64]. Including them in the model could contribute however to improve theoretical predictions for phytoplankton distributions.

In conclusion, the model presented in this paper showed to be a valid candidate to predict the spatio-temporal dynamics of vertical chlorophyll distributions in a marine ecosystem. Although the model was devised and calibrated for oligotrophic marine waters, the analysis performed could be applied to other contexts with different levels of eutrophication, allowing to extend the theoretical investigation to marine sites close to the coast. A further extension of this model could be the inclusion of zooplankton populations and higher trophic levels in view of reproducing the seasonal dynamics of fish species [22]. As a final remark we wish to underline that the studies devoted to model and reproduce the dynamics of phytoplankton populations are of paramount importance, in view of predicting the effects of global warming on these microorganisms, and devising strategies to prevent the decline of the primary production with the consequent decrease of fish species [8, 17, 89].
